# Synthetic data augmentation for improving performance in deep learning models for anatomical landmark localization on the distal upper limb

**DOI:** 10.3389/fbioe.2026.1852405

**Published:** 2026-06-16

**Authors:** Prathiksha Padmanabha, Kasunika Guruge, H. M. K. K. M. B. Herath, Seongeun Lee, Nuwan Madusanka, Hi-Joon Park, Chang-Su Na, Myunggi Yi, Byeong-il Lee

**Affiliations:** 1 Industry 4.0 Convergence Bionics Engineering, Pukyong National University, Busan, Republic of Korea; 2 Division of Smart Healthcare, College of Information Technology and Convergence, Pukyong National University, Busan, Republic of Korea; 3 Digital Healthcare Research Center, Pukyong National University, Busan, Republic of Korea; 4 College of Korean Medicine, Kyung Hee University, Seoul, Republic of Korea; 5 College of Korean Medicine, Dongshin University, Naju-si, Republic of Korea

**Keywords:** anatomical landmark localization, deep learning, metahuman-rendered synthetic images, synthetic augmentation, traditional eastern medicine

## Abstract

Precise anatomical landmark localization is critical for the therapeutic efficacy of Traditional Eastern Medicine, yet manual localization methods show inter-practitioner variability exceeding 5 mm. Deep learning can automate this process, but data scarcity limits model development. MetaHuman-rendered synthetic images offer a scalable alternative, though their texture fidelity and architecture-specific training effects remain underexplored. Six landmark localization models (HRNet-W32/W48; YOLO26-pose: small/medium/large/extra-large) were trained for five anatomical landmarks on the distal upper limb under two configurations: real-only (2,963 images) and real–synthetic mixed (3,863 images). Synthetic image quality was assessed via Local Binary Pattern (LBP) chi-squared distances and Gray-Level Co-occurrence Matrix (GLCM) features. All models were evaluated on 50 held-out real images from 18 participants using localization error (mm) and mean Average Precision (mAP). Real-to-synthetic LBP chi-squared distances (0.0155 ± 0.0042) fell within natural real-image variation (0.0107 ± 0.0117), confirming sufficient texture fidelity. Mixed training reduced the mean localization error across all six models. Under subject-level clustered bootstrap analysis (n = 18 participant clusters, 10,000 iterations), statistically significant improvements were observed in YOLO26x-pose (17.8%; p = 0.002), YOLO26s-pose (14.5%; p = 0.001), and HRNet-W48 (6.3%; p = 0.020). However, because the mixed training set contains approximately 30% more images and gradient updates than the real-only set, contributions from increased training volume, optimization steps, and synthetic data content cannot be fully disentangled. Detection confidence remained stable across conditions. All models achieved sub-3.1 mm accuracy under mixed training, approaching expert-level consistency. These findings offer practical guidance for integrating MetaHuman-rendered synthetic images into deep learning pipelines for Traditional Eastern Medicine applications when real data is scarce.

## 1 Introduction

Precise identification of anatomical landmarks on the human body is fundamental to Traditional Eastern Medicine, which has been practiced for over three millennia. The therapeutic stimulation of specific anatomical points modulates physiological functions and restores homeostatic balance (White and Ernst, 2004; Vickers et al., 2018). Therapeutic efficacy is fundamentally contingent upon localization accuracy; studies demonstrate that millimeter-scale deviations significantly diminish treatment outcomes and compromise inter-session reproducibility (Zhang et al., 2014; Godson and Wardle, 2019). Despite the widespread global adoption of Traditional Eastern Medicine as a therapeutic modality, anatomical landmark localization remains predominantly dependent on manual techniques involving visual inspection, palpation of anatomical landmarks, and proportional measurement systems such as the *cun* (body inch) method, which divides body segments into standardized units derived from patient-specific proportions (Lim, 2010). These subjective approaches are inherently susceptible to considerable inter-practitioner variability, with differences frequently exceeding 5–10 mm even among practitioners following standardized anatomical guidelines (Godson and Wardle, 2019; Malekroodi et al., 2024).

Deep learning has transformed medical imaging analysis in recent years, achieving human-level diagnostic performance across a wide range of clinical tasks (Lecun et al., 2015; Esteva et al., 2017; Rajpurkar et al., 2018). High-Resolution Networks (HRNet) have demonstrated particular efficacy in anatomical landmark detection by preserving spatial fidelity throughout network depth through parallel multi-resolution feature streams (Sun et al., 2019). Our previous work demonstrated that HRNet-W48 achieves strong performance for hand anatomical landmark localization using the MetaAcuPoint synthetic dataset (Seo et al., 2024; Guruge et al., 2025). More recently, the YOLO (You Only Look Once) architecture family has been extended to keypoint detection, with YOLO26-pose offering single-stage end-to-end inference with NMS-free (Non-Maximum Suppression) processing suitable for real-time clinical deployment (Redmon et al., 2016; Sapkota et al., 2025).

Medical AI development faces a fundamental data acquisition bottleneck. Clinical image collection requires institutional ethical approval, expert annotation by qualified practitioners, and substantial logistical resources. Critically, annotation variability introduces label noise that undermines ground truth reliability (Karimi et al., 2020). Bäumler et al. documented that experienced practitioners localize identical anatomical landmarks with mean pairwise disagreement of 4.8 ± 2.3 mm, with maximum discrepancies reaching 8.45 cm for certain points (Bäumler et al., 2012), motivating the adoption of controlled, single-annotator protocols to ensure label consistency in model development.

Synthetic data generation addresses real data scarcity through computational simulation, providing unlimited scalability, pixel-perfect annotations, controlled morphological diversity, and privacy preservation (Nikolenko, 2019; Giuffrè and Shung, 2023). Studies in general medical imaging have reported 5%–15% accuracy improvements when combining limited real clinical data with synthetic samples (Shin et al., 2018; Khosravi et al., 2024). However, synthetic data faces the well-documented “reality gap,” a distributional mismatch between simulated and real-world images that may degrade performance if synthetic images lack visual fidelity (Jakobi et al., 1995; Tobin et al., 2017). To validate synthetic image quality for anatomical landmark localization, this study employs Local Binary Pattern (LBP) texture analysis (Haralick et al., 1973; Ojala et al., 2002) to quantify photorealistic fidelity through chi-squared distances contextualized against natural real-image variation.

While our previous work demonstrated synthetic data feasibility using a single architecture on 760 images (Guruge et al., 2025), critical questions remain: Does synthetic augmentation consistently improve accuracy with limited real data (∼3,000 images)? Do benefits generalize across architecturally distinct models, particularly efficient YOLO detectors? Does MetaAcuPoint texture fidelity validate training suitability? Is synthetic utility universal or architecture-dependent?

This study addresses these gaps through a systematic, multi-architecture evaluation of six keypoint localization models across two training configurations: real-only (2,963 images) and real–synthetic mixed (3,863 images) at clinically relevant scale, combined with rigorous quantitative texture fidelity assessment using LBP chi-squared distance analysis. By comparing two architecturally distinct families (HRNet heatmap regressors and YOLO26-pose single-stage detectors) spanning a wide parameter range (10.4M–63.6M), our evaluation probes whether synthetic data benefits are universal or contingent upon model design. These results carry immediate implications for resource allocation in Traditional Eastern Medicine-oriented medical AI development and clinical deployment of automated landmark localization systems.

## 2 Materials and methods

**FIGURE 1:**
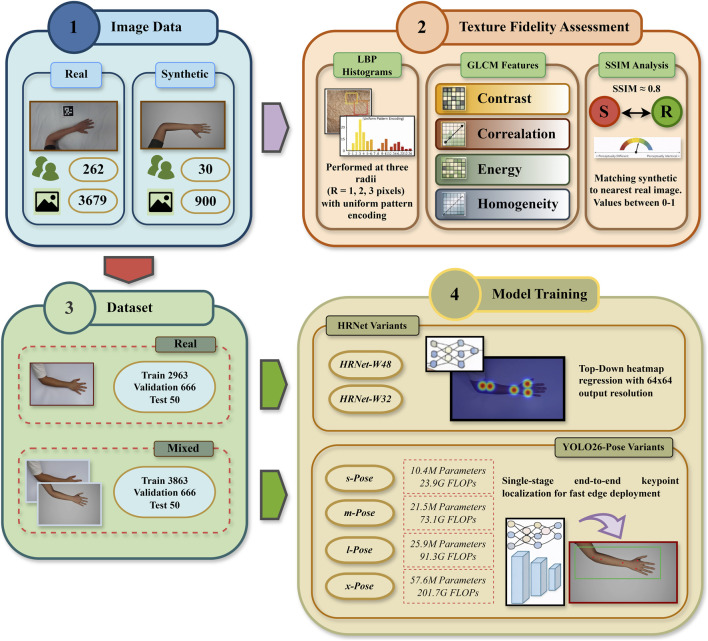
Experimental pipeline showing image data acquisition, texture fidelity assessment, dataset configuration, and model training workflow.

The complete experimental pipeline is shown in Figure 1. This study systematically evaluated six deep learning architectures for automated distal arm anatomical landmark localization across two training configurations: real-only (2,963 images) and real–synthetic mixed (3,863 images). The workflow comprised four sequential stages: dual-source data acquisition from human participants and MetaHuman-rendered synthetic avatars; quantitative texture fidelity assessment employing LBP histograms, GLCM (Gray-Level Co-occurrence Matrix) features, and Structural Similarity Index (SSIM) analysis to validate photorealistic quality of synthetic images; subject-level dataset partitioning into training, validation, and independent test sets; and systematic model training across two architectural families (HRNet variants and YOLO26-pose variants) with performance evaluation on the 50-image independent test cohort.

### 2.1 Study design

This comparative study evaluated six deep learning architectures for automated distal arm anatomical landmark localization across two training data compositions. The Institutional Review Board of Pukyong National University approved real-world image acquisition (IRB No. 1041386–202207-HR-41–02 and 2024–08-109–001). All participants provided written informed consent prior to enrollment. The study followed TRIPOD (Transparent Reporting of a multivariable prediction model for Individual Prognosis or Diagnosis) reporting guidelines for prediction model development and validation.

### 2.2 Datasets

#### 2.2.1 Real human hand dataset

We acquired 3,679 RGB images (1488 × 837 pixels) from 262 healthy adults aged 18–68 years (140 male, 122 female; predominantly East Asian), of whom 244 contributed to training and validation and 18 formed the independent test set. Images were captured under controlled conditions with participants positioning their hands flat and pronated on a white examination surface. An overhead RGB camera positioned at 60–100 cm captured top-down views under standardized lighting. ArUco fiducial markers (100 × 100 mm) placed within each frame enabled per-image pixel-to-millimeter calibration, accounting for variable camera-to-subject distances across acquisition sessions.

Landmark annotation was performed by a single trained technician selected through a rigorous multi-stage qualification process. Three candidate annotators underwent standardized training supervised by certified Traditional Eastern Medicine practitioners (Hi-Joon Park, Chang-Su Na, collectively possessing over 30 years of clinical experience). Each candidate annotated an identical 100-image calibration subset, and results were compared against expert ground-truth localizations; the technician demonstrating the highest concordance completed the full dataset annotation over a 4-month period. This single-annotator strategy was deliberately adopted to eliminate inter-rater variability, a well-documented source of label noise in anatomical landmark localization research (Bäumler et al., 2012). Annotation reliability metrics are summarized in Table 1.

**TABLE 1:** Intra- and inter-rater annotation reliability metrics.

Metric	Intra-rater	Inter-rater
Mean keypoint error	3.39 ± 2.64 mm	6.14 ± 3.51 mm
Median keypoint error	2.70 mm	5.39 mm
Bounding box IoU	0.893	0.688
Mean OKS (σ = 0.05)	0.975	0.936
% keypoints within 20 mm	99.6%	99.6%

To characterize annotation reliability, intra- and inter-rater variability were evaluated on a randomly selected subset of 50 images (250 keypoint instances). Intra-rater reproducibility was assessed through blind re-annotation by the primary annotator in a second independent session, while inter-rater reliability was assessed by a fully independent second annotator on the same images. Full reliability metrics are summarized in Table 1. Across both evaluations, 99.6% of keypoint pairs fell within 20 mm, which is within the clinically accepted cun tolerance (∼20–25 mm), supporting the reliability of the annotation protocol. Importantly, the best-performing mixed-training model (HRNet-W48; 2.81 mm) produced a localization error lower than the inter-rater disagreement observed between two human annotators on this reliability subset (6.14 mm). This comparison reflects proximity to the single-annotator reference standard rather than a direct head-to-head evaluation against human annotators. Detailed dataset statistics and demographics are summarized in Table 2.

**TABLE 2:** Real dataset statistics and demographics.

Characteristic	Value
Total images	3679
Total subjects	262
Training images	2963 (201 subjects)
Validation images	666 (43 subjects)
Test images	50 (18 subjects)
Age range	18–68 Years
Gender	140 M/122 F
Resolution	1488 × 837 pixels

**FIGURE 2:**
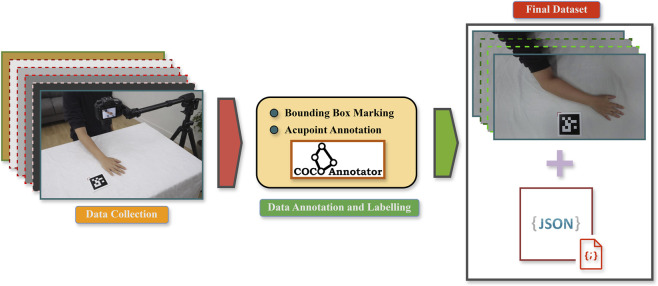
Real dataset acquisition and annotation pipeline. Data collection employed overhead RGB camera setup with ArUco markers for calibration. Annotation was performed using COCO Annotator tool in two stages: bounding box marking followed by precise anatomical landmark localization. Final dataset comprises 3,679 annotated images with ground-truth coordinates stored in JSON format.

Annotations followed the WHO Standard Acupuncture Point Locations (Lim, 2010), with five target landmarks identified in fixed sequential order: LI11 (Quchi), LI10 (Shousanli), TE5 (Waiguan), LI4 (Hegu), and TE3 (Zhongzhu). The dataset was partitioned at the participant level to prevent subject leakage: 2,963 images from 201 participants for training, 666 images from 43 participants for validation and model selection, and 50 images from 18 subsequently recruited participants, constituting a fully independent test set with no overlap in subjects, acquisition sessions, or temporal proximity (Figure 2).

#### 2.2.2 Synthetic MetaHuman dataset (MetaAcuPoint)

**FIGURE 3:**
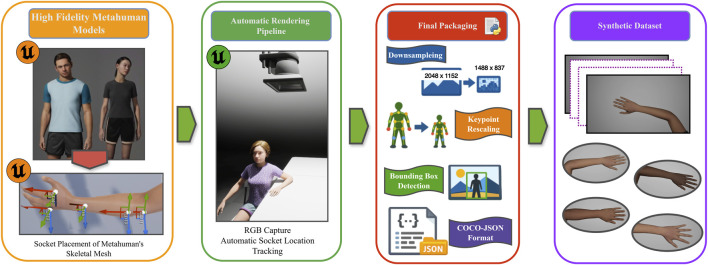
MetaAcuPoint synthetic dataset generation pipeline. High-fidelity MetaHuman models with bone-attached socket placement undergo automatic rendering in Unreal Engine 5.4, with final packaging producing COCO-JSON formatted annotations.

The MetaAcuPoint dataset comprises 900 photorealistic synthetic images generated using Unreal Engine 5.4 and MetaHuman Creator, as detailed in our previous publication (Guruge et al., 2025). The dataset incorporates 30 unique digital avatars spanning 5 skeletal types with diverse skin tones parameterized in HSV color space (Hue: 0.036–0.112, Saturation: 0.211–0.530, Value: 0.404–0.842), 15 pose variations per hand with ±10° articulation across wrist, elbow, and finger joints, and acquisition geometry matched to the real dataset in resolution, camera angle, and ambient illumination conditions (Figure 3).

Ground-truth annotations were generated through a bone-attached socket-placement procedure: a certified Traditional Eastern Medicine practitioner (Chang-Su Na) placed invisible 3D sockets at the five target landmarks on the MetaHuman skeletal mesh following WHO standardized anatomical definitions. Socket coordinates defined in bone-local space automatically maintain anatomical relationships across all avatars and pose variations.

During rendering, Unreal Engine projects these 3D socket positions to 2D pixel coordinates, providing consistent annotations across pose and avatar variations, fundamentally addressing the annotation variability problem documented by Bäumler et al. (Bäumler et al., 2012). The synthetic dataset specifications are presented in Table 3.

**TABLE 3:** MetaAcuPoint synthetic dataset statistics.

Characteristic	Value
Total images	900
Unique avatars	30 (5 skeletal types)
Poses per hand	15
Resolution	1488 × 837 pixels

#### 2.2.3 Synthetic-real mixed dataset

The combined training set comprised 2,963 real images and 900 synthetic images (total: 3,863), representing a 76.7:23.3 real-to-synthetic ratio. Training samples were randomly shuffled to create mixed mini-batches, ensuring stochastic combinations of real and synthetic examples within each batch rather than source-homogeneous batches. This approach prevents the model from learning source-specific features and encourages feature integration from both data sources.

Both training scenarios (real-only and mixed) were evaluated on the identical 50-image independent test set comprising real photographs from 18 subjects with no overlap with training participants. This held-out test set was recruited subsequently to initial data collection, maintaining strict temporal and subject-level independence to ensure unbiased performance comparability across training configurations.

It should be noted that the mixed training set contains approximately 30% more images than the real-only set. The MetaAcuPoint dataset was developed and published prior to this study (Guruge et al., 2025), and its scale (30 avatars, 5 skeletal types, 15 poses per hand) was fixed at that stage. Expanding the MetaHuman-rendered synthetic corpus further was deliberately avoided, as providing exhaustive pose variations risks the model overfitting to synthetic pose distributions rather than generalizing to unconstrained real clinical images. Because the synthetic dataset draws from only 30 avatars with 5 skeletal types, it provides limited morphological diversity compared to the 244 real participants used for training and validation. Consequently, observed improvements in the mixed condition may reflect contributions from both increased training volume and synthetic data content, and these effects cannot be fully disentangled in the current experimental design. This is discussed as a limitation in Section 4.6.

#### 2.2.4 Model architectures

We evaluated two distinct architectural families comprising six models in total. The HRNet family included width-based variants HRNet-W32 and HRNet-W48. Both HRNet models employ top-down heatmap regression with 64 × 64 output spatial resolution (Sun et al., 2019). In this two-stage approach, a separate detector first identifies the hand region via bounding box prediction, and the HRNet backbone then predicts landmark locations within detected regions through spatial heatmap generation.

The YOLO26-pose family comprises single-stage end-to-end keypoint localization models featuring NMS-free inference with Distribution Focal Loss elimination for streamlined edge deployment (Sapkota et al., 2025). We evaluated four YOLO26-pose model scales: small (s), medium (m), large (l), and extra-large (x). This experimental design enables systematic comparison of width *versus* depth scaling within the HRNet family, two-stage *versus* single-stage detection paradigms across families, and model capacity effects across the complete computational spectrum. Detailed architectural specifications, including parameter counts, FLOPs, and backbone types for all six models, are provided in Table 4.

**TABLE 4:** Model architecture specifications.

Model	Parameters (M)	FLOPs (G)	Backbone
HRNet-W32	28.5	7.1	HRNet-32
HRNet-W48	63.6	14.6	HRNet-48
YOLO26s-pose	10.4	23.9	CSPDarknet
YOLO26m-pose	21.5	73.1	CSPDarknet
YOLO26l-pose	25.9	91.3	CSPDarknet
YOLO26x-pose	57.6	201.7	CSPDarknet

#### 2.2.5 Training protocol

All models were trained using the Adam optimizer (Kingma and Ba, 2014) with an initial learning rate of 5 × 10^−4^ and cosine annealing schedule for 300 epochs with batch size 32. HRNet models employed mean squared error loss on heatmaps; YOLO26-pose models used multi-task composite loss integrating generalized IoU (Rezatofighi et al., 2019), binary cross-entropy, and Object Keypoint Similarity components. Identical data augmentation was applied across all datasets: random rotation (±30°), scaling (0.7–1.3×), translation (±20%), horizontal flip (50%), brightness/contrast jittering, and Gaussian noise (σ = 5, 30% probability). Training was conducted on NVIDIA RTX 4090 GPUs using PyTorch 2.0.1 with CUDA 12.1. Random seeds were fixed at 42 for reproducibility. The MMPose framework (MMPose Contributors, 2020) was used for HRNet implementation.

#### 2.2.6 Evaluation metrics

Primary localization accuracy was quantified as Euclidean distance error between predicted and ground-truth keypoint coordinates, converted to millimeters via per-image ArUco calibration. Overall localization error was computed by averaging across all five landmarks. Detection confidence was evaluated using mean Average Precision (mAP) at Object Keypoint Similarity (OKS) thresholds of 0.5 and 0.5:0.95 following the COCO keypoint evaluation protocol (Lin et al., 2014). For YOLO26-pose models, bounding box mAP was additionally reported. To account for within-subject correlation across the 50 test images from 18 participants, statistical inference was based on a subject-level clustered bootstrap (n = 18 participant clusters, 10,000 iterations), resampling participants rather than images. Exact p-values and 95% confidence intervals derived from the clustered bootstrap are reported for all model comparisons. As a sensitivity analysis, a linear mixed-effects model (LME) with participant as a random intercept was additionally fitted; full LME results are provided in Supplementary Table S1. Test images were selected to capture distinct poses and lighting variations for each participant, ensuring that each image represents a genuinely different clinical input rather than a redundant replicate of the same scenario.

Post-hoc power analysis (two-tailed paired t-test, α = 0.05, power = 80%, n = 50) indicated a minimum detectable effect size of Cohen’s d = 0.40, equivalent to approximately 0.74 mm given the observed within-condition variability. Under the clustered bootstrap, the three non-significant models showed improvements of 0.04–0.13 mm (d = 0.02–0.07), all of which fell substantially below this threshold, confirming that the study was underpowered to detect effects of this magnitude; these results should therefore be interpreted as inconclusive rather than evidence of no effect. Expanding to a larger and more diverse participant cohort in future work would enable more robust subject-level inference while maintaining adequate statistical power.

#### 2.2.7 Texture fidelity assessment

To quantify whether MetaAcuPoint synthetic images possess sufficient photorealistic fidelity for meaningful training augmentation, we performed systematic texture comparison between real and synthetic image populations using Local Binary Pattern (LBP) analysis (Ojala et al., 2002). Regions of interest (ROIs) were extracted as 300 × 300-pixel grayscale patches from whole images for texture descriptor computation.

LBP descriptors were computed at radius R = 2 with 16 circumferential sampling points using the uniform pattern encoding scheme. This parameterization generates rotation-invariant texture histograms that capture micro-level surface characteristics including skin texture gradients, fine-scale intensity transitions, and subsurface scattering artifacts. The uniform pattern constraint reduces histogram dimensionality from 216 to 18 bins (16 uniform binary patterns plus 2 non-uniform aggregate bins), improving statistical robustness for population-level comparisons while retaining sensitivity to dominant local texture primitives relevant to skin surface morphology.

Distributional similarity between real and synthetic LBP histograms was quantified using the chi-squared (χ^2^) distance metric, a standard non-parametric measure of histogram divergence (Haralick et al., 1973; Ojala et al., 2002). Lower χ^2^ values indicate greater distributional similarity. To establish a meaningful interpretive baseline, we computed within-class real-to-real χ^2^ distances through exhaustive pairwise comparison of randomly sampled real image subsets, thereby quantifying the natural texture variation range inherent to the real population. Real-to-synthetic distances were then contextualized against this empirical reference distribution.

**FIGURE 4:**
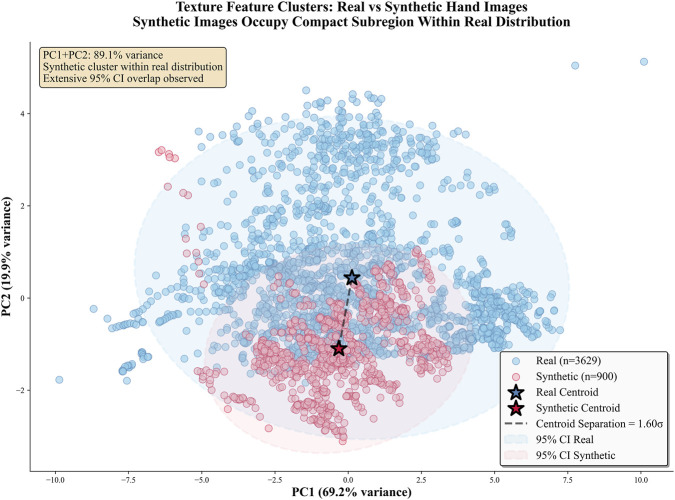
Principal component analysis of texture feature space: real *versus* synthetic hand images. PCA projection of combined LBP, GLCM, and intensity features for 3,629 real (blue) and 900 synthetic (red) images. First two components capture 89.1% of total variance (PC1: 69.2%, PC2: 19.9%). Star markers indicate cluster centroids; shaded ellipses represent 95% confidence regions. Centroid separation: 1.60σ. Extensive ellipse overlap confirms that synthetic images populate a subregion of the real image feature space.

**FIGURE 5:**
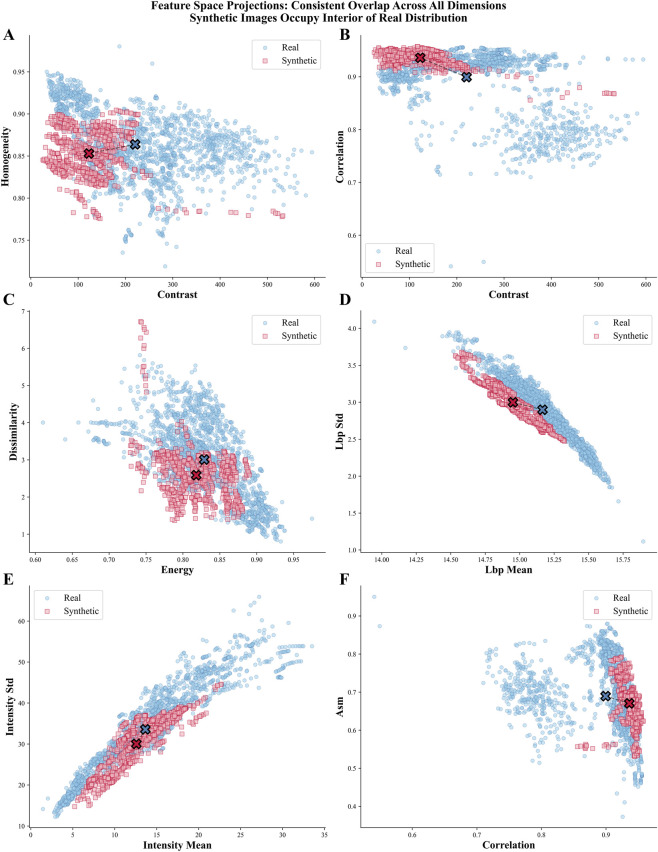
Pairwise feature space projections: real *versus* synthetic. Six selected feature pairs illustrating distributional overlap across individual texture dimensions: **(A)** Contrast vs. Homogeneity, **(B)** Contrast vs. Correlation, **(C)** Energy vs. Dissimilarity, **(D)** LBP Mean vs. LBP Std, **(E)** Intensity Mean vs. Intensity Std, and **(F)** Correlation vs. ASM. Cross markers denote population centroids. Upper row: GLCM-derived features; middle row: GLCM–LBP and LBP statistics; lower row: intensity and spatial dependency features. Synthetic images (red) consistently overlap with the denser core of the real distribution (blue), with reduced representation at distributional extremes.

**FIGURE 6:**
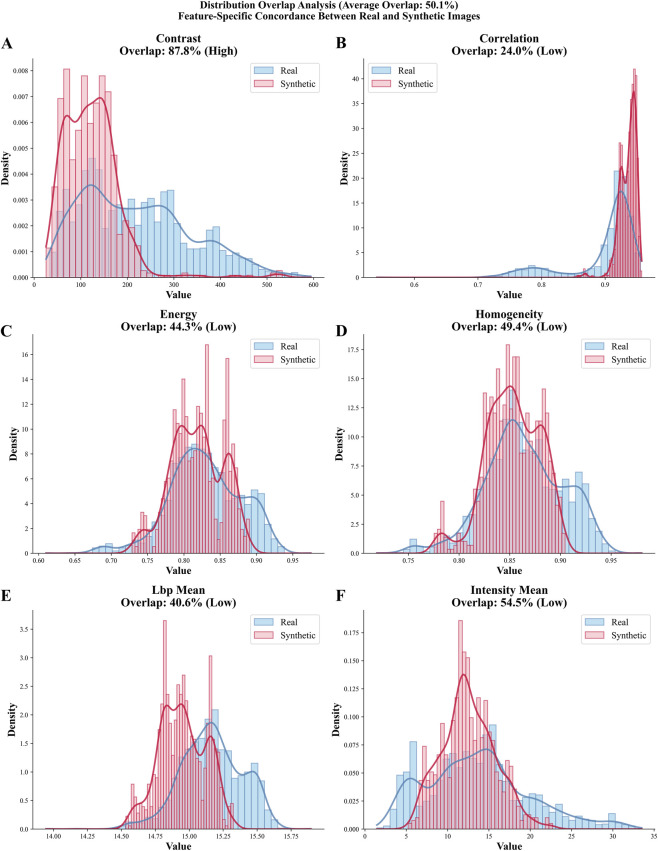
Distribution overlap analysis for six individual texture features: **(A)** Contrast (overlap: 87.8%, High), **(B)** Correlation (overlap: 24.0%, Low), **(C)** Energy (overlap: 44.3%, Low), **(D)** Homogeneity (overlap: 49.4%, Low), **(E)** LBP Mean (overlap: 40.6%, Low), and **(F)** Intensity Mean (overlap: 54.5%, Low). Kernel density estimates (curves) and histograms for real (blue) and synthetic (red) image populations. Overlap percentages quantify the area of intersection between normalized density functions. Contrast (87.8%) and Intensity Mean (54.5%) show highest fidelity; Correlation (24.0%) shows greatest divergence, indicating residual spatial dependency differences between rendered and photographed skin surfaces. Average overlap across all features: 50.1%.

Complementary analyses included GLCM features (contrast, correlation, energy, homogeneity) computed at offsets d = 1,3,5,7 pixels, and SSIM computed on 100 nearest-matched image pairs (Wang et al., 2004). Principal component analysis (PCA) was applied to a combined feature vector comprising LBP histogram statistics, first-order intensity features (mean, standard deviation), and GLCM properties to visualize the high-dimensional relationship between real and synthetic populations. Pairwise feature projections and univariate distribution overlap coefficients were computed to characterize the multi-dimensional relationship between populations (Figures 4–6).

## 3 Results and analysis

### 3.1 Texture fidelity quantification

LBP chi-squared distance analysis revealed that MetaAcuPoint synthetic images achieve micro-texture characteristics comparable to real photographs. The mean real-to-synthetic χ^2^ distance was 0.0155 ± 0.0042, falling well within the natural variation observed among real images themselves (0.0107 ± 0.0117), as summarized in Table 5. This overlap indicating that the distributional divergence between synthetic and real populations is comparable to the inherent heterogeneity within the real dataset, suggesting sufficient texture fidelity for training augmentation purposes.

**TABLE 5:** Texture fidelity metrics: real *versus* synthetic image comparison.

Metric	Real images (mean ± SD)	Synthetic images	Coverage
LBP χ^2^ distance (R = 2)*	0.0107 ± 0.0117	0.0155 ± 0.0042	Within baseline range
GLCM contrast †	220.6 ± 117.40	122.8 ± 60.90	Within real variance
GLCM homogeneity	0.864 ± 0.039	0.853 ± 0.026	Within real variance
GLCM energy	0.829 ± 0.050	0.818 ± 0.035	Within real variance
GLCM correlation	0.899 ± 0.055	0.936 ± 0.013	Within real variance
SSIM‡	1.00 (reference)	0.797 ± 0.039	Moderate similarity

* chi-squared distance between LBP, histograms at radius R = 2 with 16 sampling points (uniform patterns, 300 × 300 px grayscale); †, GLCM, properties computed at offsets d = 1,3,5,7 pixels; ‡, SSIM, computed on 100 nearest-matched pairs. Values: mean ± SD.

Principal component analysis of the combined texture feature space demonstrated overlap between real and synthetic image populations (Figure 4). The first two principal components captured 89.1% of total variance (PC1: 69.2%, PC2: 19.9%), with synthetic images (n = 900) forming a compact cluster positioned 1.59 standard deviations from the real image centroid (n = 3,629). The 95% confidence ellipses exhibited overlap, indicating that synthetic images populate a subregion of the real feature space rather than constituting a distinct distributional mode.

Pairwise feature projections across six texture dimension pairs (Figure 5) showed consistent overlap between the two populations, with synthetic images occupying the interior of the real distribution in most feature planes and reduced representation at distributional tails. Univariate distribution overlap analysis (Figure 6) revealed that the degree of real–synthetic concordance varied across individual texture features: contrast showed 87.8% overlap, while correlation exhibited only 24.0%, with an average overlap of 50.1% across all features. This indicates that the MetaHuman rendering pipeline replicates certain texture properties more faithfully than others.

The GLCM properties showed that synthetic images fell within the variance range of real images across all four Haralick features: contrast (122.8 ± 60.90 vs. 220.6 ± 117.40), homogeneity (0.853 ± 0.026 vs. 0.864 ± 0.039), energy (0.818 ± 0.035 vs. 0.829 ± 0.050), and correlation (0.936 ± 0.013 vs. 0.899 ± 0.055) (Table 5). SSIM analysis on 100 nearest-matched image pairs yielded a mean value of 0.797 ± 0.039, indicating moderate perceptual similarity adequate for augmentation purposes, though not identical to real photographs (Wang et al., 2004).

### 3.2 Overall localization performance

Table 6 presents the primary outcome: mean localization error across all six models under real-only *versus* mixed training conditions on the independent 50-image test set. Real–synthetic mixed training reduced the mean error in all six models. Under subject-level clustered bootstrap analysis (n = 18 participant clusters, 10,000 iterations), three models achieved statistically significant improvements (Figure 8): YOLO26x-pose achieved an error reduction of 0.65 mm (−17.8%; p = 0.002; 95% CI: [−1.195, −0.243] mm), YOLO26s-pose achieved an error reduction of 0.48 mm (−14.5%; p = 0.001; 95% CI: [−0.815, −0.157] mm), and HRNet-W48 achieved an error reduction of 0.19 mm (−6.3%; p = 0.020; 95% CI: [−0.362, −0.029] mm). The remaining three models showed consistent directional trends toward improvement (1.3%–4.3% reduction) that did not reach statistical significance under this conservative analysis.

**TABLE 6:** Overall localization performance on independent test set (n = 50 images; subject-level clustered bootstrap, n = 18 clusters).

Model	Real (mm)	Mixed (mm)	Δ (mm)	Change (%)	95% CI	p-value
HRNet-W32	2.92	2.88	−0.04	−1.4%	[−0.126, +0.117]	p = 0.830
HRNet-W48	3.00	2.81	−0.19	−6.3%	[−0.362, −0.029]	p = 0.020 *
YOLO26s-pose	3.31	2.83	−0.48	−14.5%	[−0.815, −0.157]	p = 0.001 *
YOLO26m-pose	3.01	2.88	−0.13	−4.3%	[−0.308, +0.054]	p = 0.187
YOLO26l-pose	3.11	3.07	−0.04	−1.3%	[−0.314, +0.244]	p = 0.740
YOLO26x-pose	3.65	3.00	−0.65	−17.8%	[−1.195, −0.243]	p = 0.002 *

Δ = Mixed − Real. negative values indicate improvement; *, statistically significant under subject-level clustered bootstrap (n = 18 participant clusters, 10,000 iterations). Figure 8 reports error reduction as Real − Mixed where positive values indicate improvement. All 95% CIs, derived from clustered bootstrap.

**FIGURE 7:**
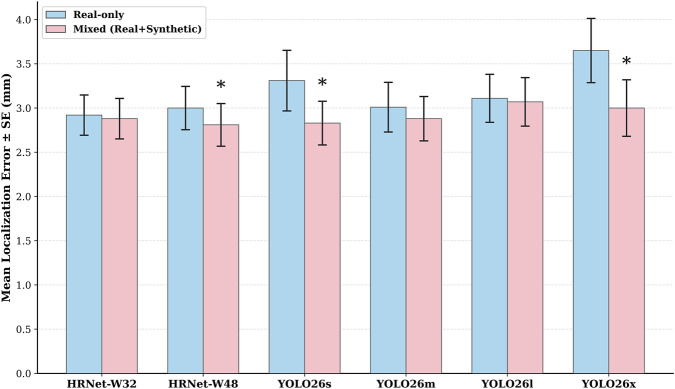
Mean localization error (±SE) for six models under real-only and mixed training conditions. Mixed (Real + Synthetic) training reduced mean error across all architectures. Asterisk (*) indicates statistically significant improvement under subject-level clustered bootstrap (p < 0.05); three models significant: HRNet-W48, YOLO26s-pose, YOLO26x-pose.

**FIGURE 8:**
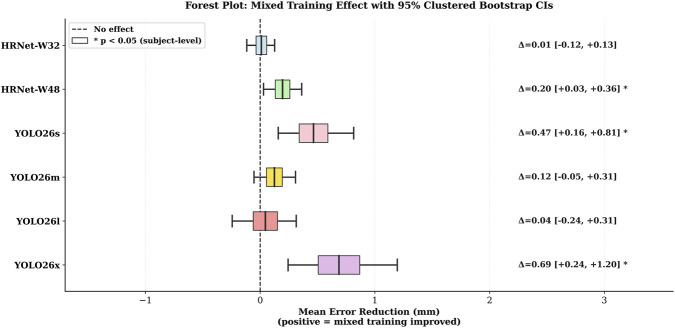
Forest plot of mixed training effect with 95% clustered bootstrap confidence intervals. Positive values indicate error reduction (mixed training improved). YOLO26s-pose, YOLO26x-pose, and HRNet-W48 achieved statistically significant improvements (p < 0.05) under subject-level clustered bootstrap, with 95% confidence intervals excluding zero. The remaining three models showed non-significant trends.

All models achieved clinically relevant accuracy under both training conditions. The worst-performing configuration (YOLO26x-pose, real-only) produced 3.65 mm error, which remains below the 5 mm threshold commonly cited as the upper bound for clinically acceptable localization error. The best configuration (HRNet-W48, mixed) achieved 2.81 mm, approaching the inter-practitioner variability documented in manual localization studies (Bäumler et al., 2012). Under mixed training, all models achieved sub-3.1 mm mean error. A comparative visualization of mean localization error across all models under both training conditions is presented in Figure 7, while the corresponding forest plot of mixed training effect sizes with 95% clustered bootstrap confidence intervals is shown in Figure 8.

### 3.3 Detection confidence and mAP performance

Table 7 presents mean Average Precision results for all six models under both training conditions. Bounding box detection was near-perfect for YOLO26-pose models (BBox mAP@0.5 = 0.995 across all variants and conditions) and consistently high for HRNet models (BBox mAP@0.5 = 0.909 across both variants and conditions), confirming that hand detection was not a limiting factor for keypoint localization accuracy. The identical HRNet BBox mAP values reflect the use of the same pre-trained detector across all HRNet training configurations.

**TABLE 7:** Detection confidence: mean Average Precision across all models.

Model	Dataset	BBox mAP@0.5	BBox mAP@0.5:0.95	Pose mAP@0.5	Pose mAP@0.5:0.95
HRNet-W32	Real	0.909	0.556	0.891	0.432
	Mixed	0.909	0.556	0.864	0.342
HRNet-W48	Real	0.909	0.556	0.915	0.438
	Mixed	0.909	0.556	0.915	0.438
YOLO26s-pose	Real	0.995	0.849	0.995	0.994
	Mixed	0.995	0.847	0.995	0.992
YOLO26m-pose	Real	0.995	0.857	0.995	0.993
	Mixed	0.995	0.851	0.995	0.992
YOLO26l-pose	Real	0.995	0.852	0.995	0.992
	Mixed	0.995	0.846	0.995	0.992
YOLO26x-pose	Real	0.995	0.850	0.995	0.992
	Mixed	0.995	0.858	0.995	0.992

mAP, mean Average Precision computed using OKS-based COCO, keypoint evaluation protocol; BBox, bounding box detection; Pose, keypoint localization. HRNet BBox, values reflect a shared pre-trained detector.

Pose mAP@0.5 was uniformly high for YOLO models (0.995 across all variants and conditions). For HRNet, Pose mAP@0.5 was 0.891 (real) and 0.864 (mixed) for HRNet-W32, while HRNet-W48 maintained 0.915 under both conditions. At the stricter OKS 0.5:0.95 threshold, YOLO models ranged from 0.992 to 0.994 with minimal variation. HRNet-W32 showed a decrease from 0.432 to 0.342 under mixed training, while HRNet-W48 remained stable at 0.438. This dissociation, whereby Euclidean error (Table 6) improved alongside reduced Pose mAP@0.5:0.95, arises from the non-linear properties of the OKS Gaussian kernel: increased spatial variance in individual predictions, even when mean Euclidean error improves, can disproportionately reduce OKS scores at strict thresholds. The absence of this dissociation in HRNet-W48 suggests it is specific to the lower-capacity variant. Overall, synthetic augmentation did not compromise detection reliability, and Euclidean error and OKS-based mAP should be interpreted as complementary rather than redundant metrics.

### 3.4 Architecture-dependent synthetic data effects

Per-landmark analysis (Table 8) revealed that synthetic data benefits were neither uniform across anatomical landmarks nor consistent across architectures. For YOLO26x-pose, mixed training improved localization for all five landmarks, with the largest gains at LI11 (4.18 mm–3.30 mm, −21.1%) and LI4 (3.43 mm–2.62 mm, −23.6%). YOLO26s-pose similarly showed broad improvement, most dramatically for LI4 (−34.9%) and TE3 (−23.2%). These two models exhibited predominantly beneficial responses to synthetic augmentation across the full anatomical range.

**TABLE 8:** Per-anatomical landmark localization error on independent real test set: Real-only *versus* mixed training comparison (mm).

Model	Dataset	LI11	LI10	TE5	LI4	TE3	Overall
HRNet-W32	Real	3.37 ± 1.86	3.73 ± 1.97	3.12 ± 1.77	2.23 ± 1.28	2.18 ± 1.17	2.92
	Mixed	3.28 ± 1.83	3.73 ± 1.64	2.95 ± 2.14	2.26 ± 1.33	2.20 ± 1.15	2.88
HRNet-W48	Real	3.53 ± 1.98	4.24 ± 2.33	2.83 ± 1.90	2.23 ± 1.32	2.14 ± 1.13	3.00
	Mixed	3.30 ± 1.95	3.74 ± 2.10	2.65 ± 2.01	2.29 ± 1.42	2.05 ± 1.04	2.81
YOLO26s-pose	Real	3.31 ± 2.32	3.44 ± 1.82	3.00 ± 2.32	3.10 ± 2.39	3.70 ± 3.26	3.31
	Mixed	2.97 ± 1.60	3.35 ± 1.81	3.00 ± 2.17	2.38 ± 1.43	2.41 ± 1.73	2.83
YOLO26m-pose	Real	3.23 ± 2.24	3.71 ± 2.28	3.28 ± 2.41	2.54 ± 1.63	2.28 ± 1.37	3.01
	Mixed	3.24 ± 1.99	3.63 ± 2.03	3.25 ± 2.08	2.23 ± 1.36	2.08 ± 1.40	2.88
YOLO26l-pose	Real	3.66 ± 2.23	3.95 ± 2.19	3.13 ± 2.05	2.31 ± 1.37	2.54 ± 1.75	3.11
	Mixed	3.97 ± 2.53	4.03 ± 2.28	2.80 ± 1.81	2.36 ± 1.65	2.20 ± 1.41	3.07
YOLO26x-pose	Real	4.18 ± 2.64	3.91 ± 1.87	3.12 ± 2.15	3.43 ± 3.11	3.59 ± 3.07	3.65
	Mixed	3.30 ± 2.42	3.46 ± 2.32	2.86 ± 2.30	2.62 ± 2.01	2.75 ± 2.24	3.00

Mixed training combines real images with MetaHuman-rendered synthetic data. All values represent localization error in millimeters on an independent test set of 50 images per landmark.

**FIGURE 9:**
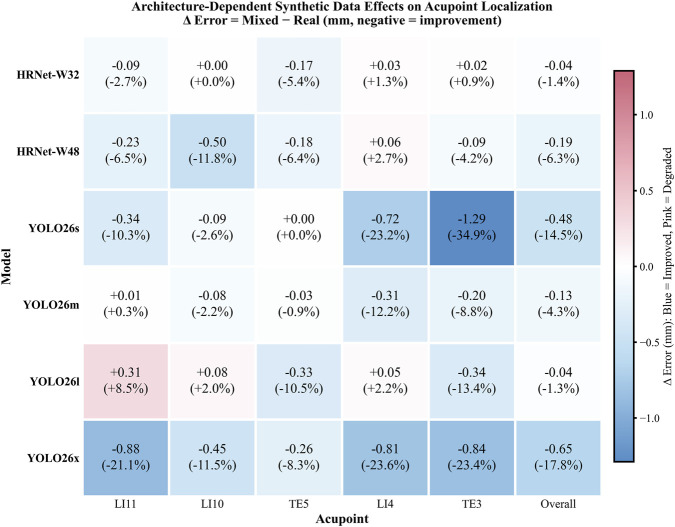
Architecture-dependent synthetic data effects on anatomical landmark localization. Each cell shows the change in mean localization error (Δ = Mixed − Real, mm) with percentage change in parentheses. Negative values (blue) indicate improvement from mixed training; positive values (pink) indicate degradation.

The pattern reversed for certain other architectures. YOLO26l-pose presented a strikingly mixed profile: mixed training degraded performance at LI11 (+8.5%), LI10 (+2.0%), and LI4 (+2.2%), while improving TE5 (−10.5%) and TE3 (−13.4%). HRNet-W32 exhibited a similarly fragmented response, modest gains at LI11 and TE5 coexisted with slight degradations at LI4 and TE3, producing a net overall improvement of only 1.4%. These per-landmark results, visualized as a heatmap of Δ error across all model × landmark combinations (Figure 9), reveal a clear pattern: smaller-capacity and single-stage models exhibit predominantly blue cells (improvement), while higher-capacity and two-stage models display more heterogeneous coloring.

Aggregating across all models under real-only training (Table 8), landmarks exhibited differential baseline localization difficulty. LI10 (Shousanli) proved most challenging (mean error: 3.83 mm), followed by LI11 (Quchi, 3.54 mm) and TE5 (Waiguan, 3.08 mm). The two hand landmarks, LI4 (Hegu) and TE3 (Zhongzhu), demonstrated the lowest errors at 2.64 mm and 2.58 mm respectively. This difficulty ranking likely reflects anatomical factors: LI10 and LI11 are forearm points defined relative to elbow flexion creases and muscle bellies, which exhibit greater morphological variability across individuals compared to the bony landmarks used to define hand landmarks.

Notably, synthetic data augmentation did not uniformly reduce errors for more difficult landmarks. While LI10 benefited under some models (YOLO26s-pose: 3.44 mm–3.35 mm; YOLO26x-pose: 3.91 mm–3.46 mm), it showed minimal or negative responses in others. This landmark-specific pattern reinforces that synthetic data benefits are mediated by architecture-dependent learning dynamics rather than simply addressing inherent landmark difficulty.

## 4 Discussion

### 4.1 Synthetic data augmentation with limited real data

The primary finding of this study is that incorporating MetaHuman-rendered synthetic images into a limited real training set reduced mean localization error across all six models evaluated. Under subject-level clustered bootstrap analysis (n = 18 participant clusters, 10,000 iterations), three models achieved statistically significant improvements: YOLO26x-pose (−17.8%; Δ = −0.65 mm; p = 0.002; 95% CI: [−1.195, −0.243] mm), YOLO26s-pose (−14.5%; Δ = −0.48 mm; p = 0.001; 95% CI: [−0.815, −0.157] mm), and HRNet-W48 (−6.3%; Δ = −0.19 mm; p = 0.020; 95% CI: [−0.362, −0.029] mm). The remaining three models showed consistent directional trends toward improvement (1.3%–4.3% reduction) that did not reach statistical significance under this conservative analysis. This provides empirical evidence that synthetic augmentation can deliver meaningful accuracy gains under data-constrained conditions (∼3,000 real images), though the magnitude and statistical reliability of improvement depend on the model architecture.

An important caveat is that the mixed training set contains approximately 30% more images (3,863 vs. 2,963) than the real-only set. Because the MetaAcuPoint dataset draws from only 30 avatars with 5 skeletal types, these synthetic images provide limited morphological diversity compared to the 244 real subjects (training and validation participants, excluding the 18-subject test set). Replacing a subset of real images with synthetic ones would reduce the representation of real anatomical variation without adding proportional diversity, making such a volume-matched design uninformative given the narrow avatar pool. Consequently, the observed improvements may reflect contributions from both increased training volume and synthetic data content, and these effects cannot be fully disentangled in the current study. Future work with substantially more diverse synthetic avatar pools may enable volume-matched experiments that isolate the specific contribution of synthetic data quality.

The texture fidelity assessment supports the quality of MetaHuman-rendered synthetic images for training purposes. The real-to-synthetic LBP χ^2^ divergence (0.0155 ± 0.0042) falls within the natural variation among real images (0.0107 ± 0.0117), and GLCM features generally fall within real image variance ranges (Table 5). However, the average univariate feature overlap of 50.1% and SSIM of 0.797 indicate that while synthetic images are adequate for augmentation, they are not identical to real photographs. The lower correlation overlap (24.0%) suggests residual differences in spatial dependency patterns between rendered and photographed skin surfaces (Wang et al., 2004; Abdusalomov et al., 2023; Pasios and Nikolaidis, 2025). Taken together, these results indicate that the MetaHuman rendering pipeline produces images of sufficient fidelity for training augmentation (Jakobi et al., 1995; Tobin et al., 2017), while acknowledging that measurable distributional differences remain.

### 4.2 Architecture-dependent response patterns

The three models showing statistically significant improvement, YOLO26s-pose, YOLO26x-pose, and HRNet-W48, span both architectural families and a wide capacity range (10.4M–63.6M parameters), suggesting that model capacity alone does not predict synthetic data benefit. The two significant YOLO26-pose models represent opposite ends of the capacity spectrum yet both benefited substantially, consistent with the single-stage paradigm creating a learning context where additional training examples alleviate multi-task competition for representational capacity. HRNet-W48 showed a smaller but statistically significant improvement (−6.3%; p = 0.020), while HRNet-W32 showed only a non-significant trend (−1.4%; p = 0.830), indicating within-family sensitivity differences. The significance of HRNet-W48 under clustered bootstrap but not under linear mixed-effects sensitivity analysis (p = 0.158) suggests this finding warrants confirmation in larger cohorts.

**FIGURE 10:**
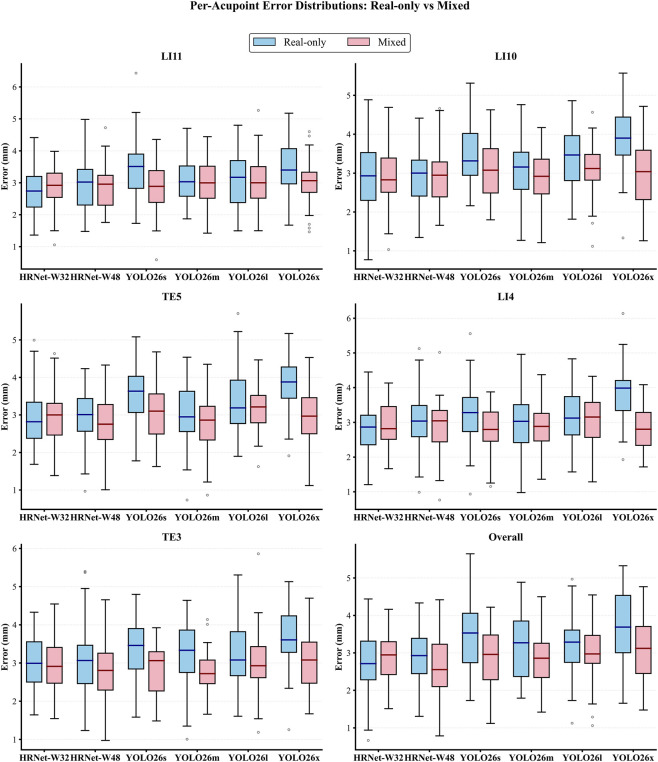
Per-landmark error distributions: real-only *versus* mixed training. Boxplots show error distributions for each model across individual landmarks (LI11, LI10, TE5, LI4, TE3) and overall. Mixed training (pink) generally shows lower medians and tighter distributions compared to real-only training (blue), particularly for YOLO26s-pose and YOLO26x-pose.

Per-landmark analysis (Table 8; Figures 9, 10) further illustrates this heterogeneity. YOLO26x-pose improved across all five landmarks, while YOLO26l-pose exhibited a mixed profile, with degradation at LI11 and LI10 alongside improvement at TE5 and TE3, indicating that synthetic data effects depend on the interaction between model architecture and anatomical target rather than providing uniform benefit.

The mAP results (Table 7) provide additional context. Bounding box detection was consistently strong across all models and conditions (BBox mAP@0.5: 0.909–0.995), and pose mAP remained largely stable, suggesting that synthetic augmentation primarily affects spatial localization precision rather than overall detection reliability. The exception is HRNet-W32, which showed a decrease in Pose mAP@0.5:0.95 (0.432 → 0.342) despite improved Euclidean error. This dissociation arises from the non-linear properties of the OKS Gaussian kernel: increased spatial variance in individual predictions, even when mean Euclidean error improves, can disproportionately reduce OKS scores at strict thresholds. The absence of this dissociation in HRNet-W48 suggests it is specific to the lower-capacity variant. Euclidean error and OKS-based mAP should therefore be interpreted as complementary rather than redundant metrics.

### 4.3 Clinical implications for Traditional Eastern Medicine

From a clinical standpoint, all six models achieved sub-3.1 mm accuracy under mixed training, and even the worst-performing real-only configuration (YOLO26x-pose at 3.65 mm) remained well below the 5 mm threshold commonly cited as the upper bound for clinically acceptable localization error (Sherman et al., 2005; Godson and Wardle, 2019). These accuracies approach and in some cases surpass the inter-practitioner variability of 4.8 ± 2.3 mm documented by Bäumler et al. (2012), suggesting that automated systems are reaching a consistency comparable to experienced practitioners.

For resource-constrained deployment in clinical or educational settings, YOLO26s-pose offers perhaps the most attractive profile: 10.4M parameters, 2.83 mm mean error under mixed training, and NMS-free single-stage inference amenable to edge devices. Its 14.5% improvement from synthetic augmentation means that a modest investment in MetaHuman rendering can meaningfully boost accuracy without requiring expensive real data expansion. Where computational resources are less constrained, HRNet-W48 at 2.81 mm achieves marginally lower error, though with 63.6M parameters and two-stage inference overhead that may be impractical for real-time applications.

### 4.4 Comparison with prior work

Our findings extend the existing literature on synthetic data for anatomical landmark localization in several ways. Sun et al. (2025) reported a 3.2% accuracy improvement using their AcuSim dataset for cervicocranial acupoints in a single-architecture evaluation. By contrast, our multi-architecture comparison reveals that the benefit of synthetic augmentation ranges from 1.4% to 17.8% depending on the model, a variation masked by single-architecture studies. Yang et al. (2025) achieved 4.2 mm accuracy for back acupoints using a real-only deep learning approach; our best mixed-training result (2.81 mm) represents a substantial improvement, though differences in anatomical region and dataset composition caution against direct comparison. The purely synthetic approach of Hasan et al. (2024) for hand keypoint detection yielded 6.8 mm error, considerably higher than our mixed-training results. This gap underscores the value of real data anchoring: MetaHuman-rendered synthetic images alone lack the distributional richness needed for accurate generalization, but when blended with even a modest real dataset, they provide complementary morphological coverage that meaningfully improves performance.

### 4.5 Annotation reliability

Our single-annotator strategy for the real dataset was deliberately adopted to eliminate inter-rater variability, a practical performance ceiling given the 4.8 ± 2.3 mm disagreements documented between experienced practitioners (Bäumler et al., 2012). The single annotator was selected for maximal concordance with expert ground truth through a formal qualification process, providing internally consistent labels. Synthetic annotations, generated via bone-attached socket projection, provide consistent coordinates across all avatar and pose variations. The mixed dataset thus combines internally consistent human labels with geometrically consistent synthetic labels, a quality profile that may itself contribute to the observed training benefits.

Intra- and inter-rater reliability were formally evaluated on 50 randomly selected images (250 keypoint instances). Intra-rater error was 3.39 ± 2.64 mm (OKS: 0.975; IoU: 0.893), whereas inter-rater error was 6.14 ± 3.51 mm (OKS: 0.936; IoU: 0.688), approximately 1.8× higher, consistent with variability reported in medical imaging annotation tasks. The best-performing mixed-training model (HRNet-W48; 2.81 mm) produced a localization error lower than the inter-rater disagreement observed between two human annotators on this reliability subset (6.14 mm). This comparison reflects proximity to the single-annotator reference standard rather than a direct head-to-head evaluation against human annotators, and suggests that the automated system achieved clinically consistent localization performance within the variability range of expert annotations.

### 4.6 Limitations

Several limitations should be acknowledged. First, the MetaAcuPoint dataset draws from only 30 unique avatars with 15 fixed poses per avatar, constraining the morphological and postural diversity of synthetic training examples relative to the 262 real participants spanning unconstrained natural poses. Expanding future synthetic corpora to include broader avatar morphologies and pose ranges would better support volume-matched experimental designs that isolate the independent contribution of synthetic data quality from training volume effects. Second, the mixed training set contains approximately 30% more images than the real-only set, and because both configurations used identical hyperparameters (300 epochs, batch size 32), the mixed condition received approximately 30% more gradient updates (∼36,000 vs. ∼27,600 steps). Consequently, observed performance gains may reflect contributions from increased training volume, additional optimization steps, and synthetic data content, none of which can be fully disentangled in the current design. To address within-subject correlation across the 50 test images from 18 participants, a subject-level clustered bootstrap (n = 18 clusters, 10,000 iterations) was adopted as the primary statistical analysis; a linear mixed-effects sensitivity analysis with participant as a random intercept confirmed the two primary findings (YOLO26s-pose and YOLO26x-pose), though not HRNet-W48, which should be interpreted with caution. Furthermore, *post hoc* power analysis confirmed a minimum detectable effect size of Cohen’s d = 0.40 (approximately 0.74 mm) at 80% power with n = 50; the three non-significant models showed improvements of 0.04–0.13 mm (d = 0.02–0.07), falling substantially below this threshold and should therefore be interpreted as inconclusive rather than evidence of no effect. The current test set size was constrained by ethical clearance limitations on real participant recruitment; future work should validate these findings on a larger and more diverse independent test cohort to improve statistical power and strengthen generalizability claims. Third, the evaluation is restricted to five anatomical landmarks on the distal upper limb captured in top-down views, and generalizability to other anatomical regions, viewpoints, or landmark systems remains to be established. The predominantly East Asian composition of the real dataset may further limit generalizability across populations with different skin tones and limb morphologies; prospective validation on ethnically diverse cohorts is warranted. Our texture fidelity assessment focuses on micro-texture statistics (LBP, GLCM, SSIM) and does not evaluate higher-level semantic properties such as anatomical consistency or pose plausibility. Architectural coverage (10.4–63.6M parameters) does not extend to very small or very large models, leaving open questions about capacity-data matching at the parameter extremes. Finally, two additional training configurations, synthetic-only training and synthetic pretraining with real fine-tuning, were not evaluated here, as the primary objective was to assess augmentation utility under realistic clinical conditions where some real data is always available. These represent important future directions that would directly quantify synthetic-to-real generalizability and provide practical strategies for data-scarce clinical deployment.

## 5 Conclusion

This study demonstrates that MetaHuman-rendered synthetic images, assessed through texture analysis as having sufficient fidelity for training augmentation, can reduce deep learning anatomical landmark localization error when combined with limited real training data. Across six architectures from two families evaluated on an independent test set, mixed real–synthetic training reduced localization error by 1.4%–17.8%. Under subject-level clustered bootstrap analysis (n = 18 participant clusters, 10,000 iterations), three models achieved statistically significant improvements: YOLO26x-pose (−17.8%; p = 0.002), YOLO26s-pose (−14.5%; p = 0.001), and HRNet-W48 (−6.3%; p = 0.020). Importantly, because the mixed training set contains approximately 30% more images than the real-only set and received approximately 30% more gradient updates (∼36,000 vs. ∼27,600 steps), the relative contributions of increased training volume, optimization steps, and synthetic data content cannot be fully disentangled, and observed gains should be interpreted accordingly. Single-stage models (YOLO26s-pose, YOLO26x-pose) benefited most, while HRNet-W32 showed a non-significant trend (−1.4%; p = 0.830). Under mixed training, all models achieved sub-3.1 mm mean error, falling below the inter-rater disagreement observed between two human annotators on the reliability subset (6.14 mm). This comparison reflects proximity to the single-annotator reference standard rather than a direct head-to-head evaluation against human annotators. YOLO26s-pose (10.4M parameters, 2.83 mm error, NMS-free single-stage inference) offers a practical choice for resource-constrained clinical deployment. Detection confidence (mAP) remained stable across training conditions, and Euclidean error and OKS-based mAP should be interpreted as complementary rather than redundant metrics. These findings provide practical guidance for integrating MetaHuman-rendered synthetic images into Traditional Eastern Medicine-oriented deep learning pipelines: when real data is scarce, pairing efficient single-stage architectures with synthetic augmentation may yield the largest gains. Future work should extend this evaluation to broader anatomical regions, larger test cohorts, and more diverse synthetic avatar pools to better isolate the contribution of synthetic data quality from training volume effects.

## Data Availability

The raw data supporting the conclusions of this article will be made available by the authors, without undue reservation.
