# Supplementary material for: Synthetic data augmentation for improving performance in deep learning models for anatomical landmark localization on the distal upper limb
Source: Front Bioeng Biotechnol. 2026 Jun 16;14:1852405. doi: 10.3389/fbioe.2026.1852405 (PMC13314767; doi:10.3389/fbioe.2026.1852405)
Supplement: Supplementary file 1 [file Table1.docx]

**Supplementary Table S1** Sensitivity analysis: linear mixed-effects model results for localization error (mm) under mixed versus real-only training. Fixed effect: training condition (mixed = 1, real-only = 0). Random effect: participant intercept. n = 50 images, 18 participants.

| **Model** | **β (mm)** | **SE** | **95% CI** | **p-value** | **Sig.** | **Interpretation** |
| --- | --- | --- | --- | --- | --- | --- |
| HRNet-W32 | −0.041 | 0.103 | [−0.244, +0.161] | 0.689 | NS | No significant effect |
| HRNet-W48 | −0.189 | 0.134 | [−0.452, +0.074] | 0.158 | NS | No significant effect |
| YOLO26s-pose | −0.488 | 0.181 | [−0.842, −0.133] | 0.007 | * | Significant improvement |
| YOLO26m-pose | −0.123 | 0.141 | [−0.399, +0.152] | 0.381 | NS | No significant effect |
| YOLO26l-pose | −0.044 | 0.192 | [−0.420, +0.333] | 0.820 | NS | No significant effect |
| YOLO26x-pose | −0.645 | 0.214 | [−1.065, −0.226] | 0.003 | * | Significant improvement |

*β = regression coefficient for training condition; negative values indicate lower error (improvement) under mixed training. SE = standard error. *, p < 0.05; NS, not statistically significant. Models fitted using restricted maximum likelihood estimation (REML) with participant as random intercept (n = 18 clusters). YOLO26s-pose and YOLO26x-pose remain significant under LME; HRNet-W48 does not reach significance (p = 0.158), consistent with near-zero between-participant variance detected in convergence diagnostics.*
